# The Punishment of the Insane

**Published:** 1899-09-16

**Authors:** 


### THE PUNISHMENT OF THE INSANE.

So great was the horror excited in the public mind by the revelations which were made, during the earlier parts of this century, as to the cruelties which used to be perpetrated upon lunatics both within and outside asylums; so complete was the reaction in all medical writings against the barbarities which formed part of the "treatment" of these unfortunates in days of old; and so general has been the acceptance of kinder methods, that a whole generation has grown up soaked in a feeling of abhorrence at the very idea of punishment of any sort as applied to the insane. The same feeling has been accentuated in no small degree by much that has been taught, and by still more that has been suggested, as to the irresponsibility of those suffering from mental disease; and although the more extreme views held by some have never received general acceptance, and although the judges have always remained clear-headed—pig-headed, some people call it—on the subject, yet the public, seeing the "plea of insanity" successfully urged as between a man's crime and his punishment outside the asylums, have not been slow to assume that all kinds of punishment for faults committed within an asylum must be wrong, and, therefore, must be things of the past under the humane system now in vogue.

To the common man who looks upon disease as an entity, who persists that a person must be either sane or insane, either responsible or irresponsible, there is so great a spice of absurdity in the idea of letting a man off for murder on the plea of irresponsibility, and then when he gets into an asylum punishing him for shouting in the passages, that it seems incredible that such things should happen. Yet without some system of punishment how is order to be maintained in our great asylums? Thus it happens that notwithstanding the mighty sway and the general acceptance of the humaner methods of these latter days we still find alienists and asylum managers discussing the question of punishment in asylums. As is well known, there are some who say that punishment is not permissible, and there are many asylum managers who not only entirely avoid the use of any kind of correction which could inflict pain, but whose aim and pleasure is to be able to go further and to say in their annual report that "no restraint and no seclusion were employed during the past year."

No doubt these gentlemen are perfectly in order, and punishment in the sense of causing pain is never used; but if we consider the bearing of this question of punishment of the insane upon the problem of their responsibility, we find much to show that lunatics are affected by punishment much in the same way as other people, although in different degree; and if we look under the surface we find that even at the best asylums some system of punishments exists. The lash, the chain, and the cell, and all the horrors have been long abolished, but a new form of discipline has come into being which may be termed "punishment by deprivation." Lunatics are now surrounded by many comforts which are not necessary, but the deprivation of which, although it does not "hurt," does, so far as the mind is concerned, act directly as a punishment; they are also given many pleasures which, although not statutory, are of the greatest possible service to them in relieving the tedium of their long confinement. These things are of the greatest possible service also to the superintendent, for they put in his hands a useful and handy form of punishment, although only "punishment by deprivation." By many alienists these forms of punishment are glossed over as "methods of treatment," "withdrawal of privileges," "aids to self-control," &c., but they are really punishments, given as such, accepted as such, and acting as such in preventing the repetition of offences. Unless the definition of punishment is to be seriously narrowed, it is hard indeed to distinguish between a system of government by the "bestowal and withdrawal of privileges" from one by a "system of rewards and punishments." The proved efficacy of this system, however, brings us at once face to face with the question of irresponsibility, for if the lunatic is sufficiently conscious of the "rightness" or "wrongness" of his actions to be kept under control by this artificial class of punishment, why should he escape thenormal consequences of his acts? Dr. Thomas Drakes,* writing on this subject, quotes the words used by Mr. Justice Wright at Warwick in 1892, where he said, "the responsibility of an accused person must depend upon the answer which must be given to the question, 'Could he help it?'" Taking this criterion as a test, he says that insane patients can and do help doing wrong when appropriate measures are taken to prevent their misconduct, and he asks why should not such means be employed in every such case where conciliatory means fail to effect any improvement?

* *Journal of Mental Science*, July, 1899.

